# High strength films from oriented, hydrogen-bonded “graphamid” 2D polymer molecular ensembles

**DOI:** 10.1038/s41598-018-22011-7

**Published:** 2018-02-27

**Authors:** Emil Sandoz-Rosado, Todd D. Beaudet, Jan W. Andzelm, Eric D. Wetzel

**Affiliations:** 0000 0001 2151 958X grid.420282.e U.S. Army Research Laboratory, Aberdeen Proving Ground, MD 21005 United States

## Abstract

The linear polymer poly(p-phenylene terephthalamide), better known by its tradename Kevlar, is an icon of modern materials science due to its remarkable strength, stiffness, and environmental resistance. Here, we propose a new two-dimensional (2D) polymer, “graphamid”, that closely resembles Kevlar in chemical structure, but is mechanically advantaged by virtue of its 2D structure. Using atomistic calculations, we show that graphamid comprises covalently-bonded sheets bridged by a high population of strong intermolecular hydrogen bonds. Molecular and micromechanical calculations predict that these strong intermolecular interactions allow stiff, high strength (6–8 GPa), and tough films from ensembles of finite graphamid molecules. In contrast, traditional 2D materials like graphene have weak intermolecular interactions, leading to ensembles of low strength (0.1–0.5 GPa) and brittle fracture behavior. These results suggest that hydrogen-bonded 2D polymers like graphamid would be transformative in enabling scalable, lightweight, high performance polymer films of unprecedented mechanical performance.

## Introduction

Two-dimensional (2D) materials such as graphene and molybdenum disulfide have extraordinary intrinsic stiffness and strength^1,2^, but low fracture toughness because of their brittle nature^3,4^. Low fracture toughness for these materials persists from monolayer through multilayer^5,6^ due to extremely weak inter-layer van der Waals (vdW) interactions, observed in graphite as early as 1928 by Sir William Bragg in his seminal work on crystallography^7^. Because 2D vdW solids have flaw-sensitivity and low shear strength through all length scales, they have yet to find relevance as stand-alone structural materials. Instead, they are best suited for use as solid lubricants^8^.

The emergence of 2D polymers^9,10^ provides a new opportunity to design a two-dimensional molecule that forms high strength, hydrogen-bonded solids. Previously proposed 2D polymers have not been designed for inter-molecular hydrogen bonding. Notable examples include covalent organic frameworks (COFs) with hydrazone^11^ linkages that form *intra*-molecular hydrogen bonds; or amide-linked, sparse COFs that would likely form a low density of hydrogen bonds^12^. Additionally, because most COFs are designed with a relatively sparse covalent network, in many cases to achieve tailored porosity for selective chemical separation, their intrinsic molecular strength and stiffness will be lower than denser molecular networks such as graphene.

Our new molecule is inspired by commercial high performance polymer fibers, which are among the stiffest and strongest engineering materials per unit mass available today^13^. One particular class of materials, poly(p-phenylene terephthalamide) (also called para-aramids or PPTA), is frequently used in these applications under the tradenames Kevlar and Twaron. These PPTA fibers possess a unique combination of high stiffness and strength with resistance to elevated temperature, chemicals, ultraviolet, moisture, and creep^14^. This performance is enabled by two key features of the polymer: i) a rigid aromatic backbone, and ii) strong intermolecular hydrogen bonding^15^. Aromaticity provides stiffness and strength to the molecule, while hydrogen bonding allows for macroscopic strength and stiffness at relatively modest molecular weights^16^. Intrinsic stiffness and hydrogen bonding also encourages liquid crystalline behavior in solution, leading to spontaneous alignment that simplifies the scaled manufacturing of oriented, chain-extended fibers^17,18^.

Analogous to the highly-aligned, chain-extended linear polymers of PPTA fibers, we propose to create an ensemble of finite molecular weight, aligned, “plane-extended” two-dimensional para-aromatic polyamide molecules, called “graphamid.” Like PPTA solids, strong hydrogen bonding between graphamid molecules generate high ensemble strength and stiffness at molecular weights that should be achievable via scalable polymer synthesis techniques. Unlike PPTA, however, we will show that all individual sheets in graphamid are bonded either covalently or by hydrogen bonds, leading to superior predicted strength and stiffness. Furthermore, the 2D nature of the graphamid bond network gives it strength and stiffness in all in-plane directions, unlike the unidirectional properties of oriented linear PPTA. Finally, we demonstrate that hydrogen bonding in graphamid leads to superior fracture toughness compared to vdW solids like graphite.

## Pristine monolayer structure and mechanical properties

**Figure 1:**
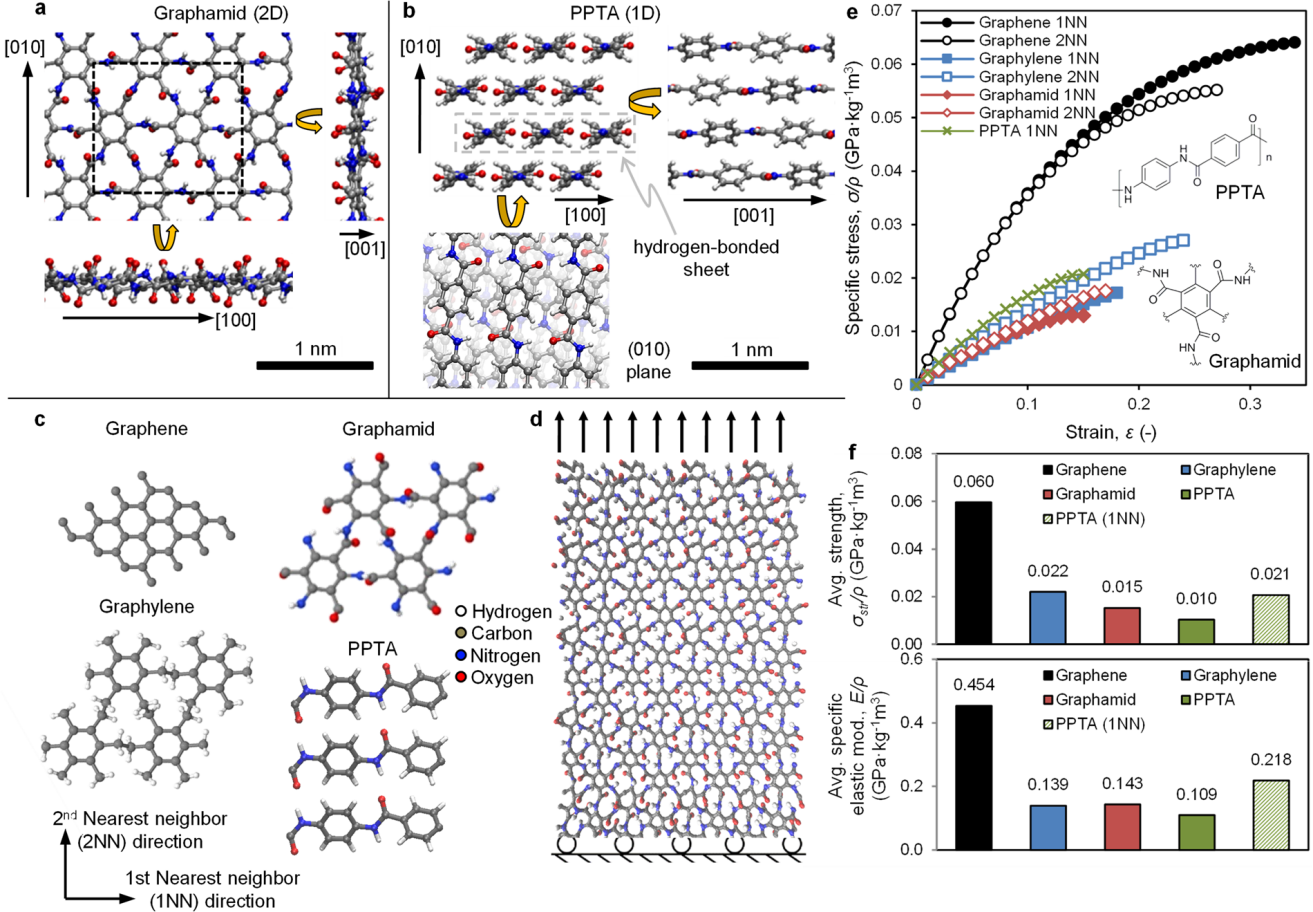
Molecular depiction of (**a**) a graphamid monolayer and (**b**) PPTA crystal structure shown in three perspectives, with the unit cell of graphamid depicted by the black dashed box, and the dashed grey box indicating a single hydrogen-bonded sheet of PPTA. C, N, O, H are colored gray, blue, red and white respectively. (**c**) Neighbor directions for graphene, graphylene, graphamid and PPTA molecules used in the DFT simulations of uniaxial tension with (**d**) a depiction of monolayer graphamid under uniaxial stress in the 1NN direction and (**e**) a plot of the specific engineering stress and engineering strain for the four materials with chemical drawing inlays, and (**f**) summary of the specific mechanical properties averaged across both 1NN and 2NN directions. “PPTA” properties refer to the isotropically averaged mechanical properties within a hydrogen-bonded sheet.

Our ensemble mechanical properties depend, in part, on the intrinsic strength and stiffness of the comprising molecules. Density functional theory simulations (DFT, see Methods section) were used to predict the lowest energy configuration of graphamid, as shown by the 72 atom rectangular primitive cell (Fig. 1a). Graphamid consists of C_6_ aromatic rings interconnected in six directions by amide bridges to form a 2D covalent polymer. In contrast, PPTA, the inspiration for graphamid, has aromatic rings linked in two opposing directions by amide bridges, forming long linear polymeric chains (Fig. 1b).

We compare the behavior of individual layers of graphamid, graphene, and “graphylene”, a recently proposed 2D polymer comprising benzene rings linked by linear ethylene^19^ (Fig. 1c). Multilayer graphylene is also a vdW solid, but has higher in-plane molecular fracture toughness than graphene due to its flexible ethylene linkers. DFT uniaxial tension simulations (Fig. 1d) were performed with loading along the first-nearest neighbor (1NN) and second-nearest-neighbor (2NN) directions (Fig. 1c). Normalizing the resulting monolayer force-per-unit-width by the areal density of the molecular sheet yields specific engineering stress (density-normalized) (*σ*_*ρ*_) versus engineering strain (*ε*) responses of the four materials (Fig. 1e). In each direction, we fit this stress-strain data to a second-order expansion of strain energy density^1,20,21^ using least-squares to determine graphamid’s density-normalized elastic modulii:

1 $${\sigma }_{\rho }={E}_{\rho }\varepsilon +{D}_{\rho }{\varepsilon }^{2},$$

where *E*_*ρ*_ is the specific elastic modulus and *D*_*ρ*_ is the 2^nd^ order, density-normalized, non-linear elastic modulus.

Figure 1f provides the resulting single molecule, directionally-averaged, density-normalized mechanical property data for graphamid, graphene, and graphylene. Graphene and graphamid demonstrate reasonably isotropic elastic responses (only showing deviation between 1NN and 2NN stress-strain responses above 10% strain), while graphylene has lower stiffness along the 1NN direction. The elastic isotropy of graphamid is related to its structure, in which the benzene rings equilibrate to tilt angles that are reasonably balanced with respect to in-plane directions. In contrast, graphylene’s stable structure exhibits preferential benzene tilting along the 1NN direction^19^, leading to a lower elastic response as those rings rotate during 1NN loading. Overall, graphene is intrinsically stiffer and stronger than graphylene or graphamid molecules. As will be show in subsequent sections, however, this advantage is not enough to overcome the weak intermolecular bonding that limits the strength of graphitic ensembles.

DFT calculations were also performed for perfectly crystalline PPTA. The strength and stiffness of PPTA along the chain direction (1NN) is higher than the values for graphamid. For uniaxial loading applications, such as ropes and cables, linear PPTA oriented along the 1NN direction would be a more ideal molecular structure than graphamid. However, for applications in which multi-axial loading is expected, such as membrane and barrier applications, isotropically averaged properties are more relevant. The PPTA crystallite arranges into hydrogen bonded “sheets”, which are themselves bonded via weaker van der Waals bonds^22^. For our PPTA calculations, we define the 2NN direction in the plane of the hydrogen-bonded sheets. Uniaxial strength and stiffness in this direction are still less than 5% of the values of the 1NN, covalently bonded direction. Therefore, in Fig. 1f, the plotted isotropic PPTA values are equal to ½ of the 1NN directional strength and stiffness values. In this isotropic comparison, graphamid is 50% stiffer and 31% stronger than PPTA per unit mass. Therefore, for film applications with multi-axial loading, the graphamid molecule is fundamentally more efficient than linear PPTA.

## Crystallinity and mechanical properties of bilayer stacks

**Figure 2:**
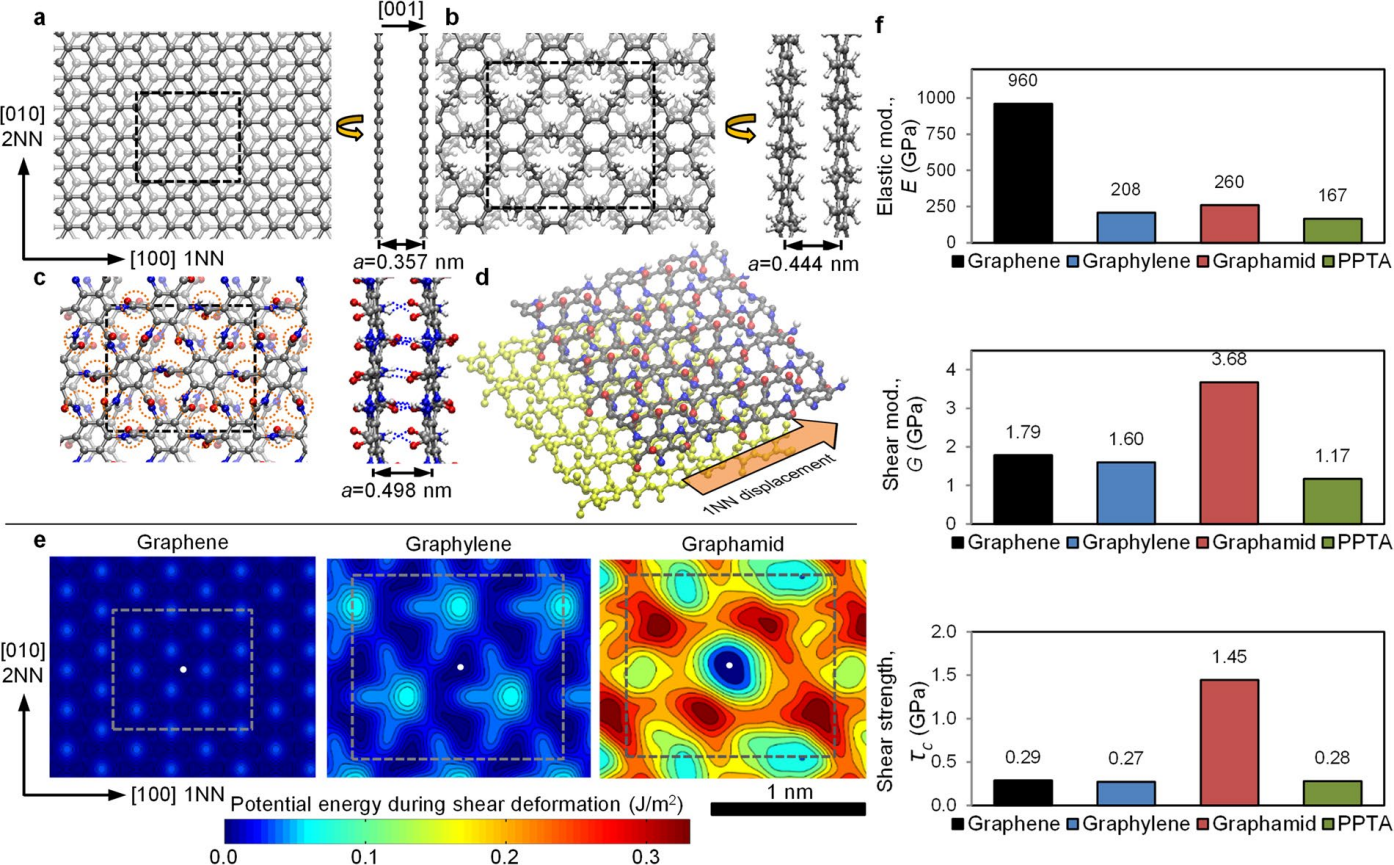
Equilibrium bilayer structures for (**a**) graphene, (**b**) graphylene, and (**c**) graphamid with C, N, O and H colored gray, blue, red and white respectively with primitive cells shown as black dashed boxes, inter-layer hydrogen bonds in graphamid are depicted as blue dashed lines, their locations are shown with orange dashed circles. Potential energy was calculated by displacing one layer with respect to another, or inducing shear deformation, as shown by (**d**) a graphamid bilayer with yellow atoms indicating the stationary bottom layer, and the top layer being displaced in the 1NN direction. Crystal directions and lattice vectors are analogous for all three structures. (**e**) Bilayer shear potential energy of graphene, graphylene and graphamid. The dashed lines correspond to the unit cells in (**a**–**c**) with the white dots are the origin of the shear displacements and also indicate the center of their respective unit cells at the equilibrium position. Lattice vector directions are shown at the lower left and have lengths corresponding to the sides of the dashed boxes for each system respectively. (**f**) Average elastic modulus and shear properties for graphene, graphylene, graphamid and PPTA.

The behavior of molecular ensembles will be strongly dependent on inter-molecular shear behavior. To determine these properties, bilayer molecular stacks were simulated in DFT for graphene, graphylene, and graphamid. System energies were calculated at various layer-to-layer relative displacements, with the lowest energy bilayer configurations shown in Fig. 2a–c. Simulations of five molecular layers show similar stacking schemes (Supplemental Section S4), indicating that the bilayer system is representative of many-layer behavior.

The graphene bilayer prefers to orient the center of one benzene ring over a carbon atom in the second layer. For graphylene, each benzene ring aligns with the interstitial gap formed by the ethylene bridge units in the next layer. Graphamid prefers to stack with a slight offset along the 1NN direction, due to the strong inter-molecular hydrogen bond interactions between amide bridge units. Graphene is the most tightly spaced, due to the perfect planarity of the molecule. Graphamid and graphylene have similar intermolecular spacing, but the density of graphamid (1.82 g/cm^3^) is significantly higher than that of graphylene (1.49 g/cm^3^) due primarily to the presence of oxygen in the graphamid structure. Crystalline spacing in PPTA were also calculated (Fig. 1b), resulting in lattice constants which match closely with experimental values for PPTA, and provide some validation of the computational techniques (Supplemental Table S5).

Using these density values, and monolayer molecular property data (Fig. 1), we can calculate the area-normalized mechanical properties for single crystals (Supplemental Tables S3). These calculations assume that layer-to-layer interactions do not appreciably affect molecular strength and stiffness for flaw-free materials, which was confirmed by running similar DFT calculations on multi-layer stacks (Supplemental Section S4). Crystalline graphene is predicted to have an elastic modulus (*E*) of 960 GPa and a strength of 120 GPa, consistent with prior computational^21^ and experimental^1,23^ studies. Graphamid has a stiffness of 260 GPa and a strength of 28 GPa, compared to 208 GPa and 33 GPa for graphylene. PPTA is predicted to have an intrinsic molecular stiffness and strength along the chain direction of 334 GPa and 32 GPa, respectively, which is in reasonable agreement with prior estimations for the PPTA crystallite from theory and experiments (Supplemental Table S6).

**Figure 3:**
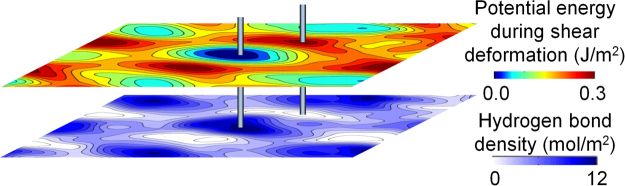
Bilayer graphamid shear potential energy as measured by displacement from the equilibrium, and corresponding inter-layer hydrogen bond density. The intersecting lines for the two surface plots indicate the shared point of lowest energy (lowest hydrogen bond density) and highest energy (highest hydrogen bond density).

To calculate intermolecular shear properties for bilayer systems of graphene, graphylene, and graphamid, layers were displaced relative to each other without rotation and then equilibrated (Fig. 2d). The shear potential energy of the system at each position is plotted in Fig. 2e, providing a visualization of the energy barriers associated with each position deviation from equilibrium (line sections of these plots can be seen in Fig. S1). Graphylene exhibits regions of higher shear potential energy compared to graphene, due primarily to the presence of larger out-of-plane deformations and stronger van der Waals interactions in graphylene. Comparatively, graphamid exhibits dramatically higher potential energy during shear deformation than graphylene or graphene. Shear modulus, *G*, is calculated by performing a parabolic fit to the energy-strain data at low strains, while shear strength,*τ*_*c*_, is calculated from the average linear slope of the full energy-strain data from minima-to-maxima. The shear properties for the 2D materials are calculated along various crystalline directions (Supplemental Table S4) and then directionally-averaged (Fig. 2f). The shear modulus of graphamid, 3.7 GPa, is over 2× higher than the shear modulus of graphene or graphylene. Shear strength for graphamid, 1.5 GPa, is 5× higher than graphene or graphylene. The superior shear behavior for graphamid is due to the strong inter-layer hydrogen bonds between molecular sheets (Fig. 3 and Supplemental Section S4). These robust hydrogen bonds are the “weakest link” of the graphamid structure, in contrast to the significantly weaker van der Waals interactions for vdW solids like graphene and graphylene.

The comparison of graphamid to PPTA is also highly favorable, and highlights a key difference between these materials. Graphamid consists of covalently bonded sheets, bridged by hydrogen bonds. PPTA, in contrast, consists of covalently bonded chains, linked within a common plane to form hydrogen-bonded sheets, which themselves are bridged by van der Waals interactions. Therefore, PPTA is likely to fail along these weak van der Waals shear planes. Calculated shear stiffness and shear strength for the hydrogen-bonded graphamid bilayer are 3× and 5× higher than the van der Waals-bonded “bilayer” of PPTA (Fig. 2f). This dramatic difference in behavior will be critical to graphamid’s superior strength and stiffness in ensemble films.

## Ensemble strength predictions

To predict the global mechanical behavior of ensembles of finite sized molecules, we follow the approach of Yoon^24^ who modeled the strength of highly oriented Vectran fibers. In this approach, the fiber is treated as an ensemble of closely packed molecules, sharing load via intermolecular forces. Because the molecules have a finite length, load is transferred from one molecule to another via shear interactions. This arrangement results in a “shear lag” effect in which the tensile load on a molecule gradually increases from zero at the molecular end point to full loading (equivalent to average ensemble tensile stress) at a finite distance from the end of the molecule. Because molecules are not being fully and uniformly loaded along their length, the global average elastic response is less stiff than the intrinsic molecular stiffness. This shear lag effect also produces intermolecular shear stresses that are maximized at the molecule ends. Ensemble failure is predicted to occur when the local shear stress at a molecular end point exceeds the intermolecular shear strength of the material. A detailed discussion of these theories, and validation of the predictions as applied to PPTA fibers, can be found in Northolt *et al*.^25^.

We can expand the Yoon theory, originally derived for linear molecular ensembles, to predict the behavior of ensembles of platelet-like 2D molecules (see Supplemental Section S5). The resulting predictions for ensemble film elastic modulus *E*_*ens*_ and strength *σ*_*ens,c*_, under conditions of perfect orientation, are:

2 $${E}_{ens}\cong E(1-\frac{\tanh (np/\delta )}{np/\delta })$$

and

3 $${\sigma }_{ens,c}\cong \alpha \frac{{\tau }_{c}}{n}(\coth (np/\delta )-\frac{1}{np/\delta }),{\rm{where}}\,n=\beta \sqrt{\frac{G}{E}},$$

where *E* and *G* are the polymer Young’s and inter-molecular shear modulii respectively, *τ*_*c*_ is the intermolecular shear strength, *p* = *L/a* is the aspect ratio of the molecule, *L* is molecular length, *a* is intermolecular distance, and *α* = *β* = 1 are pre-factors associated with the platelet geometry. The molecular cohesive parameter, *δ*, accounts for the fact that the fundamental molecular domain may not be a single molecule (*δ* = 1), but may in fact be an associated group of molecules that behave as a cohesive unit without internal shear. This molecular domain has an effective aspect ratio of *p*/*δ*. In oriented polymer fibers, this effect is observed as 1–100 nm diameter “fibrils” that emerge during fiber deformation and failure. Following Northolt *et al*.^25^, we assume the domains consist of ten cohesive molecules (*δ* = 10) for the present analysis. The precise value selected for *δ* determines a shift factor for material properties relative to absolute molecular weight, but does not have a significant effect on the comparative behavior of graphamid relative to the benchmark materials of the present study (see Supplemental Section S7).

**Figure 4:**
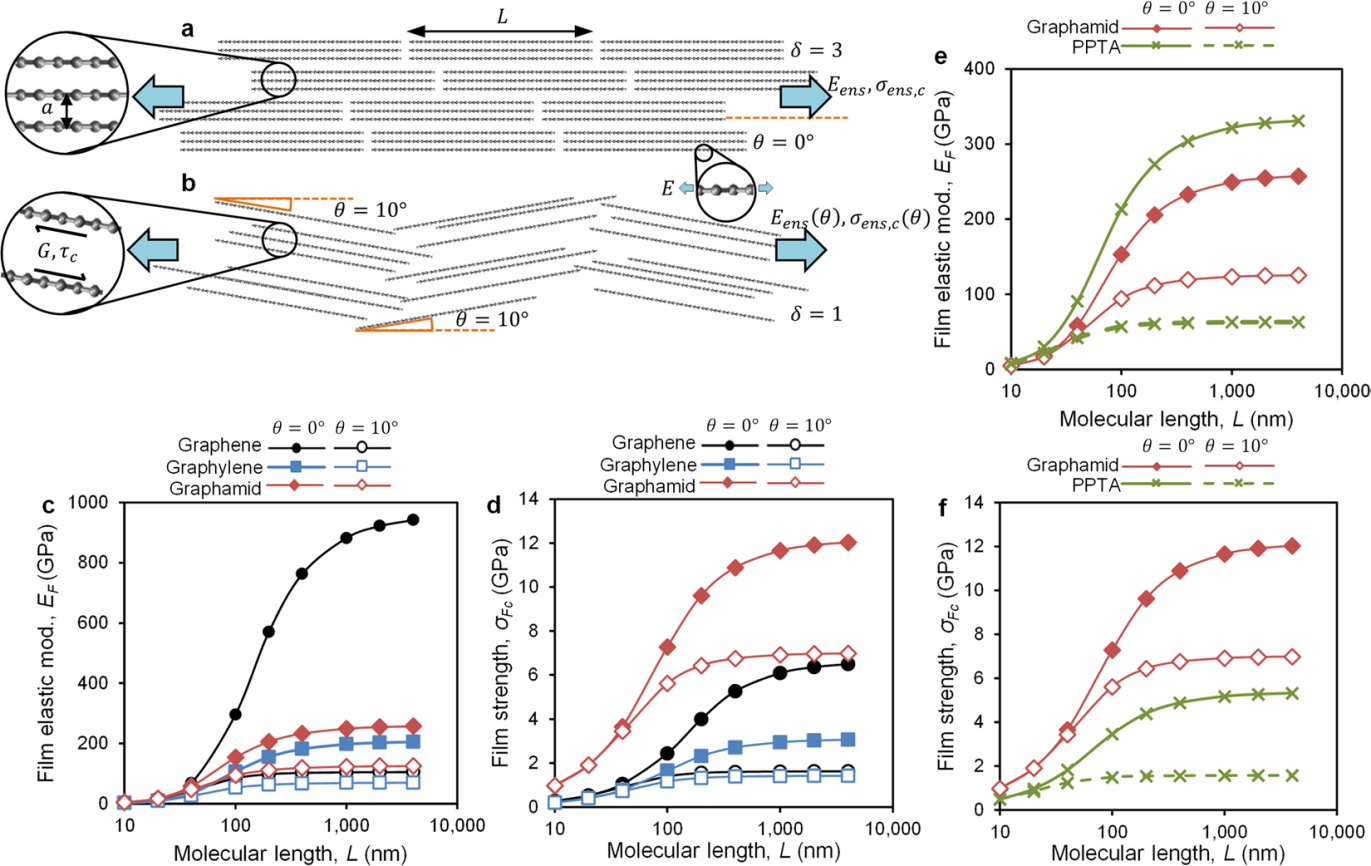
Diagram of the cross section of two monodisperse ensemble films with varying molecule angle, *θ*, and number of cohesive molecules per platelet, *δ* with (**a**) *θ* = 0° and *δ* = 3 and (**b**) *θ* = 10° and *δ* = 1, inlays show relevant geometric and material parameters. Predictions of (**c**) elastic modulus as a function of molecular length, and (**d**) strength as a function of molecular length for ensembles of graphene, graphylene, and graphamid with *δ* = 10, *θ* = 0° and 10°. Predicted ensemble (**e**) elastic modulus and (**f**) strength for graphamid film and PPTA fiber as a function of molecular length.

Using equations (2) and (3), and substituting isotropically averaged values of *E*, *G*, and *τ*_*c*_ from earlier DFT calculations (Fig. 2f), predictions of ensemble modulus and strength as a function of molecular length for graphene, graphylene, and graphamid are shown in Fig. 4c,d. For perfectly oriented molecules, graphene shows the highest modulus, followed by graphamid and graphylene. Molecular lengths of 330, 200, and 170 nm are required to achieve 75% of the asymptotic modulus values for graphene, graphylene, and graphamid, respectively, following the trend in (*G/E*) for these materials. Hydrogen bonding in graphamid reduces shear lag, increasing molecular loading at lower aspect ratios (and thus, lower molecular lengths) compared to graphene and graphylene. Strength predictions show that graphamid is expected to provide the highest strength for molecular ensembles, with values above 10 GPa possible for molecular lengths above 200 nm. Graphene and graphylene show significantly lower asymptotic strengths, due to their lower intermolecular shear strength values, and require higher molecular weights due their low shear modulus.

The modulus and strength predictions in Eqns (2) and (3) assume perfect molecular orientation. For commercial high performance fibers, perfect molecular orientation is not observed, therefore we also expect a similar lack of perfection in realistic 2D polymer ensembles (Fig. 4b). Such misorientation induces additional intermolecular shear, lessening global strength and stiffness values and magnifying the influence of intermolecular bonding. Following the approach of Northolt and Hout^26^, we estimate the in-plane modulus *E*_*ens*_(*θ*) of an ensemble film with uniform molecular orientation *θ* relative to the film plane, as

4 $${E}_{ens}(\theta )\cong {(\frac{1}{{E}_{ens}}+\frac{{\sin }^{2}\theta }{2G})}^{-1}$$

where *E*_*ens*_ is the film modulus for a perfectly oriented, finite molecular weight ensemble from Eqn. (1). For orientation effects on strength, Allen *et al*.^15^ found that, because shear failure dominates the behavior of misoriented PPTA fibers, the application of a von Mises failure model developed for off-axis loading of unidirectional composites was effective at correlating fiber strength with average molecular orientation. We apply the same approach here to estimate the effective film strength *σ*_*ens,c*_(*θ*) as

5 $${\sigma }_{ens,c}(\theta )\cong {(\frac{1}{{\sigma }_{ens,c}^{2}}{\cos }^{4}\theta +\frac{1}{{\tau }_{c}^{2}}{\sin }^{2}\theta {\cos }^{2}\theta )}^{-1/2}$$

Figure 4c,d show modulus and strength predictions for a uniform orientation angle of *θ* = 10°, within the range of *θ* = 5°−16° measured for commercial PPTA fibers of various grades^22,27,28^. Due to the very low shear modulus of graphene and graphylene, at this inclination angle graphamid exhibits the highest predicted stiffness values, reaching an asymptotic value of 126 GPa compared to 105 and 70 GPa for graphene and graphylene, respectively. For strength values, the very low shear strength of graphene and graphylene drive their mechanical strength values to less than 2 GPa, while graphamid is predicted to exceed 8 GPa strength at molecular lengths as short as 100 nm. These predictions show that the presence of hydrogen bonding in graphamid provides significant benefits in terms of strength and stiffness in molecular ensembles, and suggests that molecular weights of a few hundred nanometers should be sufficient to achieve films of outstanding strength and stiffness.

Graphene papers are ensemble membranes that have been synthesized, but have relatively low strength compared to graphamid due to their lack of hydrogen bonding (see Supplemental Section S7). In fact, the low strength of graphene papers (highest reported of 0.3 GPa^29^) relative to our theoretical strength predictions (1–2 GPa), are suggestive that poor orientation, wrinkles, lattice defects, or other factors have significantly limited the performance of these materials. A comparison of graphamid relative to graphene oxide (GO) papers and composites, functionalized for enhanced intermolecular bonding, is provided in a later discussion.

We can also use these theories to compare the behavior of graphamid with linear PPTA. Equations (2)–(5) can be applied to linear polymer ensembles by using parameters for rod-like constituent molecules, with *α* = 2 and *β* = 1.762 (the original Yoon solution, see Supplemental Section S5). Figure 4e,f compare predicted behavior for graphamid and PPTA. For PPTA predictions, we use the 1NN value for *E* (334 GPa, to replicate oriented commercial fiber behavior), shear parameters from Fig. 2f, and assume *δ* = 10. The stiffness and strength ranges predicted for PPTA are comparable to experimental values for commercial PPTA fibers with average molecular length above 100 nm^15,27,28^, providing confidence in the theory and calculations (Supplemental Figs S5–S7). The results predict that, at equal molecular length and at equal molecular misorientation (10° which is typical for commercial PPTA), graphamid exhibits significantly higher stiffness than PPTA, and 2–5× the strength over a range of molecular lengths seen in commercial PPTA fiber. This behavior is a direct result of the hydrogen bonds that bridge molecular planes in graphamid, unlike the weak shear planes that exist between hydrogen-bonded sheets in PPTA. Furthermore, the graphamid strength and stiffness values would be observed isotropically in an ensemble film, whereas the present PPTA predictions correspond to the behavior of a highly oriented fiber. For a planar PPTA with random in-plane molecular orientation, the effective isotropic film properties would be ½ the values shown in Fig. 4e,f, doubling the performance advantage of graphamid for film and membrane applications.

## Fracture of multilayer stacks

We performed atomistic fracture simulations for multilayer domains of graphene, graphylene and graphamid to demonstrate that inter-layer attraction leads to a fundamental increase in fracture toughness for graphamid relative to vdW solids. Classical molecular dynamics (MD) using the ReaxFF potential (see Methods section) was used to simulate uniaxial tension on graphene, graphylene and graphamid in the following configurations: i) a monolayer (1L) with a mode-I crack, and ii) a three layer (3L) stack with a mode-I crack in the middle layer (Fig. 4a–c). MD predictions for in-plane elastic modulus and ultimate tensile strength for a flaw-free graphamid monolayer were in good agreement with DFT calculations (Supplemental Fig. S10), indicating that the ReaxFF potential is suitable for predicting elastic and fracture properties of graphamid.

**Figure 5:**
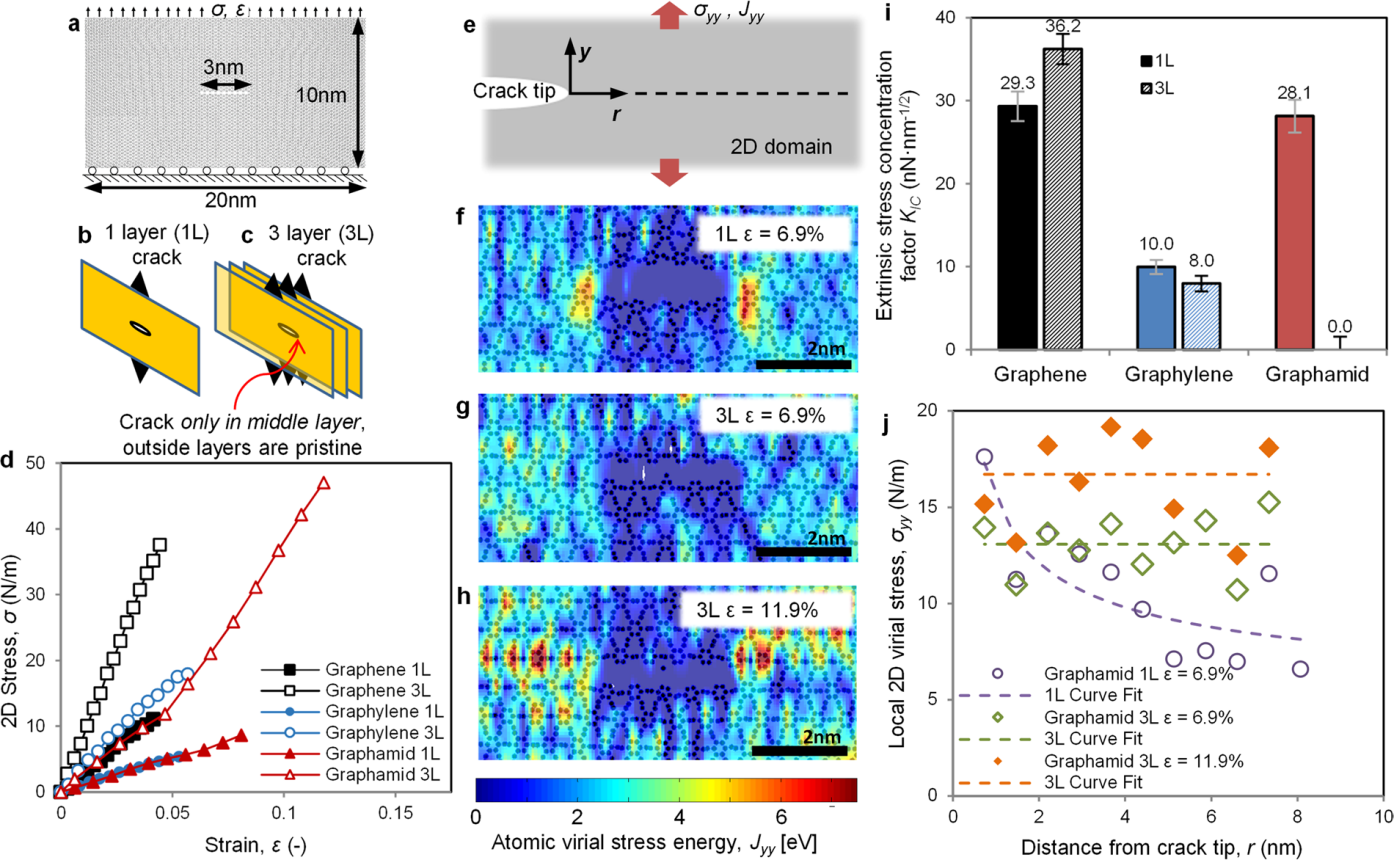
(**a**) Domain of 2D material with a pre-crack and boundary conditions, (**b**) one layer (1L) crack configuration, (**c**) three layer (3L) configuration with the pre-crack only in the middle layer and (**d**) comparison of *y*-component engineering stress/strain response for 1L and 3L graphene, graphylene, and graphamid, all lines terminate at the initiation of crack growth. All cracks are oriented in the first nearest neighbor (1NN) direction, with strain applied perpendicular to the crack at the edge of the boundary. (**e**) Depiction of the pre-crack tip and coordinates for obtaining stress profile (along the dashed line) and stress concentration factor. Atomic virial stress energy (non-volume-normalized virial stress), in the y-direction for graphamid (**f**) 1L just prior to crack propagation at strain ε = 6.9%, (**g**) middle layer of 3L at ε = 6.9%, (**h**) middle layer of 3L just prior to crack propagation at strain ε = 11.9% with C and N atoms overlaid as black circles. (**i**) Extrinsic stress concentration factor for the pre-crack in 1L and the middle layer of 3L for graphene, graphylene and graphamid (solid bars are 1L, dashed bars are 3L), (**j**) the virial stress in graphamid 1L and the middle layer of 3L as a function of distance from the crack tip, dashed lines show curve fits to Eqn. (6).

The stress-strain response for pre-cracked 3L domains are compared to 1L domains with the same area and pre-crack length for graphene, graphylene and graphamid in Fig. 5d. While graphene has the highest fracture stress for 1L, graphamid has an energy to fracture, *U*_*fracture*_, 44% larger than that of graphene, and 124% higher than that of graphylene (*U*_*fracture*_ = $(A/2)\int {\sigma }_{2D}d\varepsilon$, where *A* is domain area with a value of 10 nm × 20 nm, *σ*_*2D*_ is 2D stress and *ε* is strain). When examining 3L fracture, not only does graphamid have the highest fracture stress, it has an energy to fracture 1.8× higher than graphene and 2.8× higher than graphylene. While the 1L fracture energy of graphamid is the highest of the three materials, the 3L fracture energy of graphamid is disproportionately higher than 3L graphene and graphylene due to multilayer enhancement from hydrogen bonding. This enhancement is best seen by the increase in strain to failure of graphamid which goes from a value of *ε* = 8.1% in 1L to *ε* = 11.8% in 3L, meanwhile graphene and graphylene fail at nearly the same strain in the 1L and 3L configurations, around *ε* = 4% and *ε* = 5% respectively. The results for comparison of stress, strain and energy to fracture for the different scenarios is summarized in Supplemental Table S9.

To provide insight on the mechanism for the multilayer enhancement of fracture toughness in graphamid, an extrinsic stress concentration factor, *K*_*IC*_, was calculated for the 1L domains, as well as in the *middle layer only* for the 3L domains. The middle layer in the 3L fracture configuration was examined for stress concentration because the outer two layers are pristine, and this extrinsic stress concentration factor can provide a quantification for the reduction in stress localization due to load-sharing between layers. Local *y*-component stress, *σ*_*yy*_, is plotted as a function of distance from the crack tip, *r*, from the crack to the edge of the domain as seen in Fig. 5e. Stress is obtained from the atomistic simulation by area-averaging the atomic virial stress energy (non-volume-normalized virial stress), *J*_*yy*,_, in rectangular bins, and the resulting stress profile is used to calculate *K*_*IC*_ according to the following equation:

6 $${\sigma }_{yy}=\frac{{K}_{IC}}{\sqrt{2\pi r}}+{\sigma }_{{\rm{far}}}$$

where *σ*_*far*_ is the far-field stress. The reduction of stress concentration in 3L graphamid compared to 1L can be seen in the virial stress energy plotted in Fig. 5f–h. Graphamid 1L prior to failure at *ε* = 6.9% has a distinct stress concentration at the crack tip (Fig. 5f), while graphamid 3L at the same strain value of *ε* = 6.9% has a much more uniform stress distribution (Fig. 5 g). Even at 11.9% strain (Fig. 5 h), close to the failure strain of 3L graphamid, the stress concentration is less localized compared to the 1L sample at 6.9%. Furthermore, the shape of the graphamid crack in 1L is much more ellipsoidal, and covers a larger area than the 3L, suggesting that the outer layers are restraining the strain field around the crack in the multilayer case. The resulting stress concentration factors for 1L and 3L fracture configurations can be seen in Fig. 5i, and the stress profiles used to extract *K*_*IC*_ for all cases can be seen in supplemental Fig. S11. Both graphene and graphylene have respective stress concentration factors in 3L that are within 20% of the 1L fracture domains, while graphamid has a dramatic reduction in stress concentration factor between 3L and 1L, resulting in a stress concentration factor of effectively zero. The stress profiles for graphamid 1L at a strain of *ε* = 6.9%, 3L at *ε* = 6.9%, and 3L at *ε* = 11.9% can be seen in Fig. 5j along with their respective least-squared curve fits of Eqn. (6). While 1L has a predictable rise in stress closer to the crack tip, 3L graphamid has no perceptible increase in stress with proximity to the crack, leading to a stress concentration factor of zero at the same strain. Even at higher strain, stress does not appear to be concentrating at the crack tip compared to the edge of the boundary, although it is clear in Fig. 5h that the stress is concentrating along a line parallel to the crack, as opposed to a point near the crack tip as in the 1L case in Fig. 5f. The dramatic reduction in flaw sensitivity for 3L graphamid, compared to 1L graphamid, is a direct result of intermolecular hydrogen bonding, and suggests these materials would not exhibit the brittle fracture behavior observed in traditional vdW solids.

## Comparing graphamid with hydrogen-bonded graphitic oxide

Given the promising intrinsic mechanical properties of graphene, promoting inter-molecular bonding in graphitic ensembles is an area of active research. A common strategy is the oxidation of graphene to create epoxide and hydroxide sites for hydrogen bonding, followed by stacking into graphene oxide (GO) papers. Thus far, this approach has resulted in only modest strength achievements, reaching values of 0.03–0.3 GPa^30^. In this section, we explain why this strategy is unlikely to match the ultimate strength of graphamid ensembles.

To maximize the strength of an intermolecular interface in a GO paper, hyrdoxyl and epoxide groups on one sheet would be spatially matched with complementary epoxide and hydroxyl groups, respectively, on the adjacent sheet. Theoretically such hydrogen bonds could be packed at a density of 7 bonds/nm^2^, only a 13% lower hydrogen bond population than graphamid. In this scenario, the maximum shear strength in multilayer GO can be estimated from the hydrogen bond energy (0.32 eV)^31^, the bond distance (equilibrium at 0.2 nm, bond separation at 0.4 nm)^31^, and the ideal bond density (7 bonds/nm^2^), yielding a value of *τ*_*c,Max*_ = 1.05 GPa (see Supplemental Section S9 for full analysis). Given the approximate nature of this estimation, it is reasonable to state that such a shear strength value is comparable to our predictions for graphamid (1.45 GPa).

Unfortunately, however, experimentally realizing such theoretical intermolecular bonding densities is highly unlikely. First, GO papers are created by oxidation of graphene sheets, and experimental functionalization densities (percentage of available atomic sites that are functionalized) are typically 20–50%, with the highest reported density at 70%^32^. Through modified growth methods it was found that, due to steric exclusion, the maximum functionalization density is approximately 85%^33^. Secondly, achieving theoretical shear strengths in GO would require perfect complementary patterning of hydroxyl and epoxide groups on opposing GO faces. In reality, these functional groups populate randomly on each surface, drastically reducing the likelihood of hydrogen bond formation. In addition, GO has limited inter-layer shear strength due to self-passivation of hydrogen bonds within a given layer. One simulation study found that in multilayer GO, nearly half of hydrogen bonds are *intra*-layer bonds that do not contribute to shear strength, a result that held true even when the simulations were repeated with a reduced population of functionalization^31^. Finally, epoxides cause significant and random corrugation in GO, diminishing contact area between layers and further diminishing the likelihood of hydrogen bond formation. The combined effect of all of these factors results in a true hydrogen bond density at least 10× lower than the theoretical maximum (see Supplemental Section S9), and a commensurate order-of-magnitude loss in shear strength, leading to the poor mechanical properties observed experimentally for GO ensembles.

In contrast, graphamid has a number of fundamental advantages. First, the amide group forms hydrogen bonds with other amide groups. Therefore, there is no requirement to create matched complementary functional group patterns as is necessary with GO. Instead, simply creating self-similar graphamid molecules of good crystallinity will result in dense hydrogen bonding. For this reason, and due to the synthetic accessibility of amide chemistries, it is a preferred functional group for creating hydrogen bonded polymers^34^. Secondly, the graphamid molecule possesses these amide groups at high per-area density, and the equilibrium configuration of graphamid is effectively planar with the amide groups rotated out of plane. This molecular structure results in a high density of accessible intermolecular hydrogen bonds that would be expected to approach our theoretical predictions. Analogous advantages in PPTA - a high population of self-aligning amide groups in a “rigid rod” polymer - explain why PPTA forms high strength fibers, and why our theoretical shear strength predictions from DFT agree well with the limited experimental data available.

For these same fundamental reasons, the graphamid structure is likely to be one of the most ideal 2D polymers for creating hydrogen-bonded ensembles. For comparison, amide-containing sparse covalent organic frameworks generate *intra-*molecular hydrogen bonds, with the amide groups rotated into the molecular plane^12^. It is apparent that the tight packing of the graphamid structure forces the amide groups to rotate into a state that is amenable to intermolecular hydrogen bonding. The combination of short amide groups bridging nodal benzene rings to form a 6-fold planar covalent bond network could make graphamid an archetypal molecule for creating compact, stiff, and strong hydrogen-bonded 2D molecular ensembles.

An approach related to GO papers is to build polymer-GO composites, using a low molecular weight polymer to mediate hydrogen bonds between layers, or form direct physical crosslinks. These materials have demonstrated good toughness, but strengths remain below 0.5 GPa^35^. The same factors that limit strength development in GO papers, namely low bond density, are also likely concerns for these composites. We also note that polymer loadings in these systems are typically around 50%. In contrast, a graphamid solid does not require an intermediary polymer that would reduce the per-mass efficiency of the film, i.e. graphamid is a “100% volume fraction” reinforced material.

## Outlook

Compared to vdW solids like stacked graphene, the present analysis shows that hydrogen-bonded 2D polymers would be expected to exhibit dramatic advantages for creating films of extraordinarily high strength, stiffness, and toughness. Hydrogen bonding provides a number of key advantages. First, commercial synthesis of infinite molecular sheets of 2D polymer is likely to be exceedingly difficult. Instead, materials like graphamid will most likely be industrialized in a manner comparable to linear polymers, i.e. solution synthesis of countless finite-sized, sub-micron molecules that will then be deposited into an oriented film. The ensemble mechanical analysis demonstrates that strong intermolecular hydrogen bonding is key to achieving high strength and stiffness in such molecular ensembles. These effects become even more important as molecular orientations deviate from perfect alignment, another reality of scaled manufacturing. While this work does not consider all manufacturing-induced imperfections that can impact mechanical performance, such as contaminants, the results of neat graphamid compared to neat PPTA indicate that graphamid will be a superior mechanical material under comparable flaw conditions. Second, hydrogen-bonding between layers of graphamid provide a toughening mechanism during fracture wherein local loads are transferred to neighboring layers, diminishing stress concentration around a crack defect. Therefore, unlike vdW solids, graphamid solids should be flaw tolerant and extremely tough. Quantitatively, ensemble theory predicts that graphamid films composed with likely molecular lengths and orientations will result in films with isotropic in-plane stiffness and strength of 110 GPa and 6 GPa, and a density of 1.8 g/cm^3^, a disruptive improvement over typical high performance biaxially oriented films, or quasi-isotropic stacks of woven PPTA^20^. In fact, the strong hydrogen bonding in graphamid suggests that it will be resistant to both in-plane tension and compression, making it suitable for a wide range of structural applications. In contast, the weak van der Waals planes in PPTA fibers lead to kink band failures at low longitudinal compression loads^36^, restricting the PPTA market primarily to tensile applications such as ropes and body armor. In addition, the high stiffness of graphamid and high intermolecular interactions make it likely that it will form liquid crystalline solutions, an important characteristic for enabling scalable oriented film production. Considering the revolution in high performance materials triggered by the discovery and commercialization of Kevlar 50 years ago, and given the rapid, recent progress in COF and 2D polymer synthesis, we are hopeful that the promise of materials like graphamid generates the rapid investment and discovery necessary for a 2D polymers revolution in the 21st century.

## Methods

### Methods for first principles calculations

Throughout this study, first principles calculations are performed using Kohn-Sham density functional theory^37^ in conjunction with the widely used generalized gradient approximation exchange-correlation functional of Perdew, Burke, and Ernzerhof (PBE)^38^. The PBE functional was compared to and chosen over the Becke, Lee, Yang, and Parr^39,40^ (BLYP) functional due to higher accuracy in reproducing PPTA lattice constants with respect to the experiment^25^. Using the computational setup described below and a simulation cell of 336 atoms with dimensions ~15 × 15 × 13 Å, we find that the lattice parameters are most closely matched to experimental data for the PBE functional (Table S5); consequently PBE was chosen for all simulations.

We performed our DFT calculations using the freely available CP2K code^41^ which implements the Quick-Step method as described by VandeVondele *et al*.^42^. This methodology uses a Gaussian basis to describe the single-body functions (i.e., molecular orbitals) and a multi-grid plane wave basis for the electron density. Goedecker, Teter, and Hutter (GTH)^43,44^ pseudopotentials are used to treat the cores for carbon, nitrogen, oxygen and hydrogen. These separable dual-space Gaussian-type pseudopotentials are optimized for PBE by Krack^45^. A Gaussian basis set with triple-zeta with valence polarization functions is used for the single body orbitals, as is a plane wave cutoff of 350 Ry for the electron density. Energy self-consistence was converged to 10^−6^ Ha, forces were converged to 4.5 × 10^−4^ Ha/bohr, and stress tensor elements were converged to below 100 bar. While the structures we study are expected to be nearly charge balanced we still employ a dipole correction as described by Bengtsson^46^ to treat any serendipitous polarization due to lack of rigorous symmetry. Dispersion interactions are accounted for using a 1.5 nm cutoff with the Grimme DFT-D method with D3 parameterization as implemented in CP2K^47,48^. Results are given for the gamma point where the simulation cell contains multiples of 72 atoms depending on the number of layers studied. All first-principles calculations use periodic boundary conditions. The simulation cell lattice vectors are more than 1 nm in length and nearly orthogonal; the out of graphamid plane lattice vector was no less than 3 nm in length.

In addition to initial zero pressure graphamid structures we also computed uniaxial stress-strain curves via DFT. We treat the strain directions similar to graphene. Graphamid is analogous to graphene if the strong C_6_ graphamid rings correspond to graphene atom sites and the graphamid –CO–NH– chains correspond to graphene bonds.

### Methods for fracture simulations

Classical MD simulations were equilibrated at 0 K and subsequently integrated under constant energy (NVE) with a strain rate of 2.0 ns^−1^, similar to previous publications^19^ (see Supplemental Section S8). Simulations of GrE-2 were performed in AIREBO^49^ with a time step of 0.5 fs and a carbon-carbon interaction cutoff of 0.197 nm to avoid aphysical strain hardening, in accordance with prior literature as well as previous DFT validation for 2D polymer systems^4,19^. The unmodified ReaxFF potential^50^ was used for graphamid simulations, with a time step of 0.2 fs. Local 2D stress is determined by binning atomic virial stress energy and area-averaging the values. Atomic virial stress energy maps are performed by linearly interpolating the binned energy values.

## Electronic supplementary material

Supplementary Information
