# Supplementary material for: High strength films from oriented, hydrogen-bonded “graphamid” 2D polymer molecular ensembles
Source: Sci Rep. 2018 Feb 27;8:3708. doi: 10.1038/s41598-018-22011-7 (PMC5829261; doi:10.1038/s41598-018-22011-7)
Supplement: Supplementary file 1 — Supplementary Information [file 41598_2018_22011_MOESM1_ESM.docx]

**Supplementary Information**

**High strength films from oriented, hydrogen-bonded "graphamid" 2D polymer molecular ensembles**

Emil Sandoz-Rosado, Todd D. Beaudet, Jan W. Andzelm, and Eric D. Wetzel

U.S. Army Research Laboratory

Aberdeen Proving Ground, MD 21005

***S1. Directional mechanical property data***

Table S1 shows directional elastic parameters, stress at failure, and strain at failure, for single molecule DFT simulations of graphene, graphylene, graphamid, and PPTA subject to uniaxial tension. For single molecule calculations of graphene, graphylene, and graphamid, stresses are two dimensional, in units of N/m (calculated force normalized by the width of the calculation domain), while for PPTA chains the loads in units of N (force carried by a single chain) are reported.

To provide a more consistent basis for comparison, Table S2 normalizes each primitive mechanical property from Table S1 by its mass density (areal density for the 2D materials, linear density for PPTA). The resulting "specific" property values can be expressed as density normalized stresses (Pa / (kg/m^3^)). Effective isotropic values (directionally averaged) are calculated as the average of the 1NN and 2NN directions, which is rigorously correct for elastic modulus but an approximation for strength^1,2^. Density-normalized property values are the most rigorous basis for comparison for weight-sensitive applications such as aircraft structures and body armor.

Table S3 shows area-normalized elastic and failure properties for graphene, graphylene, graphamid, and PPTA. Material volumetric density values are calculated for graphene, graphylene, and graphamid by normalizing their molecular density values (in kg/m^2^) in Table S2 by their inter-layer spacing, calculated from DFT bilayer simulations. For PPTA, the linear molecular density value (in kg/m) in Table S2 is normalized by the unit cell area normal to the 1NN direction. Area-normalized property data in Table S3 is calculated by multiplying the density-normalized properties in Table S2 by each material's volumetric mass density value.

Table S4 shows directional intermolecular shear data for each molecule, calculated from the DFT simulations.

1. : Primitive single-molecule mechanical elastic and failure data.

1. : Density-normalized, directional mechanical elastic and failure data for single molecules. Molecular density refers to mass per unit area for graphene, graphylene, and graphamid, while referring to mass per unit length for PPTA. The molecular density values listed for the 1NN direction are also used for 2NN and isotropic property calculations.

1. : Area-normalized, directional mechanical elastic and failure data based on intermolecular spacing. The intermolecular spacing and density values listed for the 1NN direction are also used for 2NN and isotropic property calculations. Density values are volumetric mass densities.

1. : Directional intermolecular shear data. Isotropic values are the average of the directional property values. "Distance" refers to the distance from the lowest energy bilayer configuration to the position of maximum energy along a given shear direction.

***S2. Example energy versus displacement plots for shear characterization***

Example plots of strain energy and stress as a function of shear displacement, from DFT bilayer shear simulations, are shown in Fig. S1. Each color corresponds to displacements along a different shear direction.

**
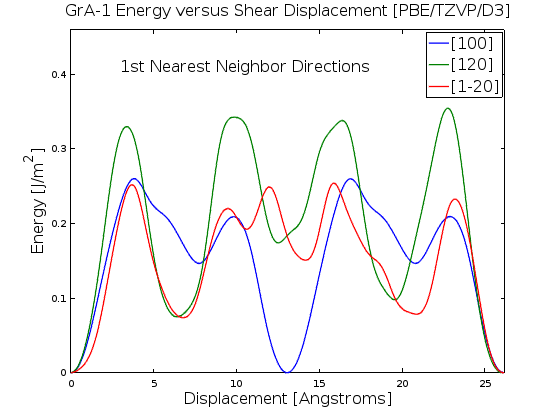

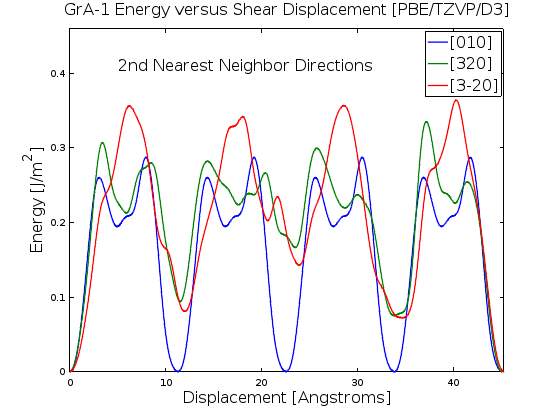
**

**(a) (b)**

**
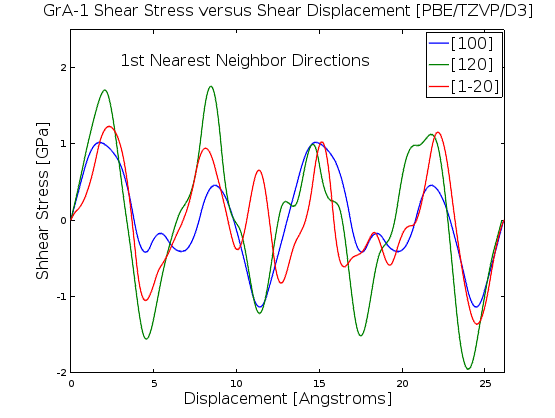

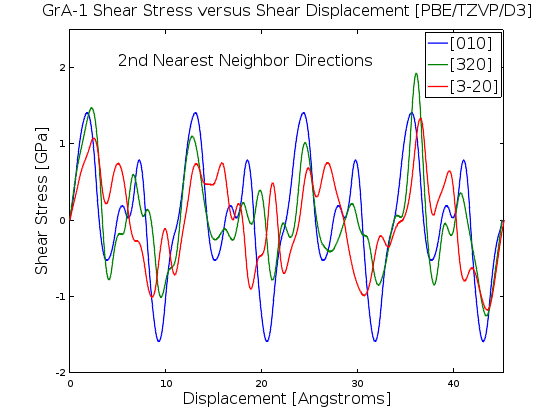
**

**(c) (d)**

1. : Graphamid shear energy along (a) 1NN and (b) 2NN directions and shear stress along (c) 1NN and (d) 2NN directions.

***S3. Validation of DFT modeling of PPTA***

This study presents the first published estimates for the structure and properties of graphamid, and therefore the results cannot be directly validated by comparison to prior experimental or computational data. However, the structure and chemistry of graphamid are very similar to PPTA, for which considerable prior literature exists. Therefore, comparing structure and properties of our atomistic predictions for PPTA with published values provides the best validation of our computational techniques and should provide reasonable confidence in the accuracy of our graphamid calculations.

***Crystalline lattice geometry and density***

Lattice parameters for the PPTA crystal have been reported by Northolt and van Aartsen (1973)^3^ as *a* = 7.87 Å, *b* = 5.18 Å and *c* (fiber axis) = 12.9 Å. Table S5 compares these values to DFT simulations using PBE and BLYP methods. While both results are reasonably accurate, the PBE lattice predictions are slightly closer to the experimental values, and are therefore used for all subsequent calculations.

We have tested the convergence of PBE results by increasing the planewave cutoff up to 1500 Ry and the size of the basis set by including additional polarization functions within the TZV2P basis set of the cp2k program^35^. The lattice vectors and angles are not affected within 0.05% and therefore we selected the computationally efficient TZVP basis set and the cutoff of planewaves at 350 Ry. We have also tested the importance of using C9 coefficients within the DFT-D3 scheme and found them to change the lattice vectors of Kevlar by less than 1%. The accuracy of our method for treating hydrogen bonded systems was also tested in other systems, such as benzamide, nomex and nylon-6-6-alpha and the experimental crystal structures were reproduced with good accuracy, similar to the one presented here for the Kevlar crystal.

Northolt and van Aartsen (1973)^3^ also used their x-ray measurements on PPTA to report a crystallographic density of 1.48 g/cc. Later measurements by Northolt and van Aartsen (1977)^4^ resulted in an estimated crystallite density of 1.525-1.545 g/cm^3^, in close agreement with our predicted value of 1.532 g/cm^3^. Note that this crystallite density is higher than the density typically measured for PPTA fibers, 1.44 g/cm^3^, likely due to the presence of voids and packing inefficiencies in a commercial polycrystalline fiber.

1. : Comparison of experimental and predicted lattice constants for the PPTA single crystal. Experimental data is from Northolt and van Aartsen^3^.

***Mechanical properties***

Table S10 compares crystallite mechanical property values from the literature. Considering experimental data, crystallite extensional stiffness has been measured either by tracking x-ray crystallite dimensions in-situ during tensile loading^5,6^, or by extrapolating fiber stiffness versus orientation data to a condition of perfect orientation^7-9^. The predicted crystallite extensional stiffness values range from 175-240 GPa. Stiffness and orientation data has also been used to estimate an inter-molecular shear stiffness, resulting in predicted values of 1.2-3.8 GPa^7-9^. Torsional single-fiber experiments were used to determine shear stiffness values of 1.8-2 GPa^10,11^. Fiber shear strength has been measured to be 0.18 GPa by Deteresa et al. (1984)^10^, while stiffness, strength, and orientation data for a wide range of fibers was used to estimate an intermolecular shear strength of 0.17 GPa by Allen et al. (1992)^9^. No experimental data has been reported on the inherent strength of a PPTA crystallite, largely because fiber strength is driven instead by intermolecular shear.

Considering theoretical estimates, Fielding-Russell (1971)^12^, Tashiro et al. (1977)^13^, and Northolt and van Aartsen (1977)^4^ used bond angle and stiffness values, combined with crystal lattice parameters, to estimate the crystallite stiffness to be 182-220 GPa. Northolt and van Aartsen (1977)^4^ used a similar approach as applied to intermolecular hydrogen bonds to estimate the shear stiffness to be 2.4-5.7 GPa. Ohta (1983)^14^ used crystalline packing information and bond strength values to estimate the crystallite strength to be 30 GPa. Termonia and Smith (1986)^15^ used kinetic fracture theory to model fiber failure, and one can use their bond activation energies to crudely estimate fracture under conditions of 0 K and very short times (see Crist (1986)^16^), resulting in a tensile strength of 24 GPa and a shear strength of 2.2 GPa. Rutledge and Suter (1991)^17^ used molecular dynamics calculations to predict a crystallite extensional stiffness of 150-350 GPa, and a shear modulus of 2.3-6.9 GPa. One study calculated a crystallite tensile strength of 1 GPa^18^, which is clearly a significant under-prediction since it falls below experimentally measured strength values for PPTA fibers, which are polycrystalline bodies limited by orientation effects, flaws, and inter-molecular shear failure. We are not aware of any direct computational predictions for intermolecular shear strength in PPTA, which is very surprising considering the critical role of this parameter in determining fiber strength.

Within this context, our predicted crystallite stiffness value of 334 GPa is around 50% higher than most prior measurements and predictions, although within range of the molecular calculations performed by Rutledge and Suter (1991)^17^. We note that both our calculations and those of Rutledge and Suter (1991)^17^ explicitly include intermolecular interactions, while more primitive earlier theoretical estimates^4,12,13^ neglect intermolecular interactions that could lead to under-prediction of crystallite modulus. Our prediction for shear modulus, 1.2 GPa, is at the lower end of the range of prior reported values. Our shear modulus is calculated for the weak plane between hydrogen-bonded sheets, whereas other shear modulus measurements and estimations may includes the effects of hydrogen-bonded planes. Our molecular tensile strength value of 32 GPa compares favorably with the estimation of Ohta (1983)^14^, and is significantly higher than the prediction of Grujcic (2011)^18^ as discussed above. Finally, our shear strength prediction of 0.28 GPa is very similar to the average of the shear strength values derived from experimental data. The shear strength estimate based on bond activation energy from Termonia and Smith (1986)^15^ is considerably higher than our calculation or other estimates, but is a very simplistic estimation and therefore is not likely to be an accurate benchmark.

Overall, the quantitative similarity of our PPTA property calculations to prior values determined via both experimental and theoretical means, provides high confidence in the validity of our atomistic calculations for PPTA. Therefore, our graphamid atomistic property calculations are also likely to be reasonably accurate.

1. : Reported molecular mechanical properties for PPTA. The upper half of the table shows values determined via experimental measurements, while the lower half of the table shows theoretical and computational modeling predictions.

***S4. Analysis of hydrogen bonding in graphamid and multilayer behavior***

The hydrogen bonding network clearly governs mechanical response of graphamid and requires more in depth analysis. The hydrogen bond (Hbond) in graphamid is formed due to an attractive interaction between a hydrogen (H) atom bonded to a donor, nitrogen (N), and an acceptor, oxygen (O), located in a different amide group. The Hbond is usually defined based on the geometric criteria; for example a Hbond is active if the bond distance between H and O is less than 2.5 Å and the N-H-O angle is at least 90° or larger. In this work we have added an energetic criteria based on the interaction energy between models of a single hydrogen bond in amide pairs represented by the trans-N-methylacetamide dimer. Extensive ab inito calculations at the MP2/aug-cc-pVDZ level identified an equilibrium configuration with H-O bond of 1.867 Å, a N-H-O angle of 169 ° and a BSSE-corrected binding energy of 10.8 kcal/mol. These results are similar to our DFT/TZVP calculations yielding an optimal H-O bond length of 1.932 Å, the N-H-O bond angle of 168.4° and the binding energy of 9.8 kcal/mol. The potential energy surface of H-O and N-H-O was scanned up to 3.6 Å and 80° respectively. We define strong hydrogen bonds in graphamid within range of 1.7 < O-H < 2.6 Å and 140 < N-H-O < 180° for the HBond distance and angle, respectively.

This analysis yields a network of hydrogen bonds because hydrogen atoms may be involved in multiple connections. The N-methylacetamide dimer model is not an appropriate approximation to model surface Hbonds because the mobility of the surface atoms forming HBonds is significantly constrained by the network of benzene rings. We have estimated the maximum energy of the intra-molecular HBonds to be 1.6 kcal/mol by scanning the O-H distance of atoms attached to the graphamid one-layer (1L) model. The strength of inter-layer hydrogen bonds for the graphamid two-layer (2L) and five-layer (5L) systems were calculated to be over 9 kcal/mol, indicating that inter-molecular hydrogen bonding is much more significant than intra-molecular hydrogen bonding. Furthermore, graphamid layers on the inside of 5L stacks did not have any detectable intra-moelcular hydrogen bonding.

Table S7 compares the number and configuration of hydrogen bonds between graphamid layers and also within the molecule. To facilitate analysis, the 5L graphamid model was divided into four sets of layers connecting layers 1 and 2, 2 and 3, and so on.

1. : Number of hydrogen bonds and their configuration for intra and inter-molecular interactions

Each external graphamid layer contains about 8 hydrogen bonds within the plane of the molecule (intra-molecular) that are much weaker than the HBonds in between graphamid molecules (inter-molecular). The graphamid layers that are on the inside of the simulated stack do not have intra-layer bonding and seem to have somewhat stronger HBond connections to their neighboring layers. This suggests that outermost graphamid layers in a stack may slide first during the shear deformation of the stack due to their weaker inter-layer bonding. The mechanism of shearing deformation involves first breaking the strong inter-layer hydrogen bonds, followed by sliding of the layers with hydrogen bonds that are intra-layer, thereby passivating the inter-layer interaction. The graphamid 2L structure with the largest shearing potential energy has about 18 intra-layer HBonds with average OH distance of 2.1 Å and N-H-O bond angle of 143°.

The mechanism of growing the graphamid molecules may also be revealed from our HBond analysis. At first, the intra-layer hydrogen bonds passivating the graphamid must be broken and the new inter-layer Hbonds need to be created. For 2L graphamid, the expense of breaking 9 intra-layer bonds is about 14 kcal/mol and it is eclipsed by a gain of about 108 kcal/mol due to formation of the inter-layer HBonds. This qualitative analysis is in good agreement with the direct prediction of the 2L graphamid formation energy calculated using a supermolecular approach. The formation energies of graphamid *per-layer* for 2, 3,4,5 and 6-layer stacks are 92.4, 101.5, 105.0, 104.7 and 106.1 kcal/mol respectively, nearly constant. Similarly, the inter-layer spacing between the graphamid layers is near constant (Fig. S2). Thus, adding a new layer of graphamid to a stack does not significantly affect the bonding characteristics of other interior layers. For graphamid with 5L or 6L, we do notice a decrease in the separation distance between inner layers. If we consider eventual batch synthesis of the graphamid systems, the intra-layer bonds, although very weak, may be useful in preventing the undesired premature association of the graphamid molecules. The graphamid molecules with passivated intra-layer hydrogen bonding may slide during flow into optimal positions when the strong inter-layer HBonds will activate, creating more stable graphamid platelets. This predicted mechanism of processing seems to be analogous to Kevlar processing conditions.

1. : Graphamid stacks with 3, 4 and 5 layers with respective inter-layer spacing

***S5. Derivation of shear lag theory for platelets***

S5.1 Strength of a polymer fiber composed of linear rigid-rod molecules: Yoon theory

Consider a single rod in an infinite polymer matrix, oriented in the direction of an applied far-field (composite) strain *ε*. Via standard shear lag theory ^19,20^ the peak shear stress *τ* occurs at the tip of the rod, and is given by

$\tau=\frac{1}{2}nE_{r}\varepsilon\tanh\left(ns\right)$ ( 7 )

where

$n^{2}=\frac{4G_{m}}{E_{r}\ln\left(P_{r}/V_{r}\right)}$ ( 8 )

and *E_r_* is rod modulus, *G_m_* is matrix modulus, *s = l / r* is rod aspect ratio, 2*l* is rod length, 2*r* is rod diameter, *P_r_* is a packing factor equal to

$P_{r}=\left\{\begin{matrix} \pi\\ 2\pi/\sqrt{3}\text{ } \end{matrix}\right.\begin{matrix} \text{for square packing} \\ \text{for hexagonal packing} \end{matrix}$ ( 9 )

where *V_r_* is rod volume fraction. Similarly, the average tensile stress acting on the rod is given by

$\sigma=E_{r}\varepsilon\left(1-\frac{\tanh\left(ns\right)}{ns}\right)$ . ( 10 )

If we assume that composite failure is driven by shear failure between the rod and matrix, then Eqn. (7) can be re-written as

$E_{r}\varepsilon_{c}=\frac{2\tau_{c}/n}{\tanh\left(ns\right)}$ ( 11 )

where *τ_c_* is the rod-matrix shear strength and *ε_c_* is the far-field strain at failure. Then, the average rod tensile stress at the moment of rod-matrix shear failure is found by substituting Eqn. (11) into Eqn. (10), yielding

$\sigma_{c}=\frac{2\tau_{c}/n}{\tanh\left(ns\right)}\left(1-\frac{\tanh\left(ns\right)}{ns}\right)$ . ( 12 )

or

$\sigma_{c}=\frac{2\tau_{c}}{n}\left(\coth\left(ns\right)-\frac{1}{ns}\right)$ . ( 13 )

where *σ_c_* is the average rod tensile stress at the moment of rod-matrix shear failure.

Now consider an oriented "rigid-rod" polymeric fiber composed of perfectly aligned, monodisperse molecules. We assume that failure of the fiber is driven by shear failure between molecules. To apply composite theory to this single fiber, we assume that the fiber "composite" consists of a molecules of stiffness *E_r_* at a volume fraction of *V_r_* = 1, with an intermolecular shear strength *τ_c_* and an effective intermolecular shear modulus of *G_m_*. In this scenario, the far-field average fiber stress is equal to the number-average of the length-averaged tensile stress within a single molecule. For a monodisperse ensemble of molecules, then all molecules have equal length-averaged tensile stresses, and the fiber strength is equal to the length-averaged tensile stress within any molecule at the point of intermolecular shear failure. In this scenario, the ultimate fiber strength *σ_fc_* is given by

$\sigma_{fc}=\frac{2\tau_{c}}{n}\left(\coth\left(ns\right)-\frac{1}{ns}\right)$ . ( 14 )

If we further consider that the fiber is entirely composed of molecules, then *V_r_* = 1 and, from Eqn. (8), assuming hexagonal packing we find that

$n=1.762\sqrt{\frac{G_{m}}{E_{r}}}$ . ( 15 )

The combination of Eqns. (14) and (15) provides a prediction for the ultimate strength of a polymer fiber with a given molecular stiffness (*E_r_*), inter-molecular shear modulus (*G_m_*), intermolecular shear strength (*τ_c_*), and molecular aspect ratio *s = l / r*. Molecular aspect ratios can be calculated based on known molecular weight and effective molecular diameter or packing density. This equation is the oft-cited fiber strength theory of Yoon ^21^. In the main body of the paper, to account for the possibility of the effective "rod" being a small number of cohesive molecules, we define an effective aspect ratio of *s* = *p/δ* where *δ* is a cohesion parameter and *p* is the aspect ratio of a single molecule.

If we further assume that the molecules are very long relative to their diameter, then *s* → ∞, and Eqn. (14) approaches the limiting behavior

$\sigma_{fc}=\frac{2\tau_{c}}{n}$ . ( 16 )

Substituting Eqn. (15) for *n* gives

$\sigma_{fc}=1.135\tau_{c}\sqrt{\frac{E_{r}}{G_{m}}}$ ( 17 )

which provides a relationship between fiber strength and molecular stiffness (*E_r_*), inter-molecular shear modulus (*G_m_*), and intermolecular shear strength (*τ_c_*).

Northolt ^22^ found that assuming that molecules behave as individual bodies tended to produce unrealistically high shear interactions; that is, the theory predicted that high strengths could be achieved at lower molecular weights than what has been observed experimentally. He attributed this discrepancy to the fact that each molecule may not behave independently; instead a finite number of molecules may behave as a cohesive body, sharing tensile stresses uniformly and not containing significant internal shear. The cohesive bodies can be thought of as the fundamental "fibrils" that comprise a fiber. In this scenario, the aspect ratio *s* is modified as

$s=\frac{\text{molecular length}}{\delta\text{ }\times\text{ }\left(\text{inter}-\text{molecular spacing}\right)}$ ( 18 )

where Northolt found that values of δ=5-20 provided reasonable approximations of observed fiber behavior ^22^.

S5.2 Strength of a polymer film composed of rigid platelet molecules: Tyson and Davies theory

Tyson and Davies ^23^ provided a platelet solution for the Cox ^19^ shear lag analysis, which we present here following nomenclature of Piggott ^20^. The peak shear stress *τ* occurs at the tip of the platelet, and is given by

$\tau=nE_{p}\varepsilon\tanh\left(ns\right)$ ( 19 )

where

$n^{2}=\frac{G_{m}}{E_{p}}\frac{V_{p}}{2-V_{p}}$ ( 20 )

and *E_p_* is platelet modulus, *G_m_* is matrix modulus, *s = l / a* is platelet aspect ratio, 2*l* is platelet length, 2*a* is platelet thickness, and *V_p_* is platelet volume fraction. Similarly, the average tensile stress acting on the platelet is given by

$\sigma=E_{p}\varepsilon\left(1-\frac{\tanh\left(ns\right)}{ns}\right)$ . ( 21 )

If we assume that composite failure is driven by shear failure between the platelet and matrix, then Eqn. (19) can be re-written as

$E_{p}\varepsilon_{c}=\frac{\tau_{c}/n}{\tanh\left(ns\right)}$ ( 22 )

where *τ_c_* is the platelet-matrix shear strength and *ε_c_* is the far-field strain at failure. Then, the average platelet tensile stress at the moment of platelet-matrix shear failure is found by substituting Eqn. (22) into Eqn. (21), yielding

$\sigma_{c}=\frac{\tau_{c}}{n}\left(\coth\left(ns\right)-\frac{1}{ns}\right)$ . ( 23 )

where *σ_c_* is the average platelet tensile stress at the moment of fiber-matrix shear failure.

Now consider an oriented "rigid-platelet" polymeric film composed of perfectly aligned, monodisperse, planar molecules. We assume that failure of the film is driven by shear failure between molecules. To apply composite theory to this film, we assume that the film "composite" consists of a molecules of stiffness *E_p_* at a volume fraction of *V_p_* = 1, with an intermolecular shear strength *τ_c_* and an effective intermolecular shear modulus of *G_m_*. In this scenario, the far-field average film stress is equal to the number-average of the length-averaged tensile stress within a single molecule. For a monodisperse ensemble of molecules, then all molecules have equal length-averaged tensile stresses, and the film strength is equal to the length-averaged tensile stress within any molecule at the point of intermolecular shear failure. In this scenario, the ultimate film strength *σ_Fc_* is given by

$\sigma_{Fc}=\frac{\tau_{c}}{n}\left(\coth\left(ns\right)-\frac{1}{ns}\right)$ . ( 24 )

If we further consider that the fiber is entirely composed of molecules, then *V_p_* = 1 and, from Eqn. (20), we find that

$n=\sqrt{\frac{G_{m}}{E_{p}}}$ . ( 25 )

The combination of Eqns. (24) and (25) provides a prediction for the ultimate strength of a polymer film with a given molecular stiffness (*E_p_*), inter-molecular shear modulus (*G_m_*), intermolecular shear strength (*τ_c_*), and molecular aspect ratio *s = l / a*. Molecular aspect ratios can be calculated based on known molecular weight and effective molecular diameter or packing density.

If we further assume that the molecules are very long relative to their diameter, then *s* → ∞, and Eqn. (24) approaches the limiting behavior

$\sigma_{Fc}=\frac{\tau_{c}}{n}$ . ( 26 )

Substituting Eqn. (25) for *n* gives

$\sigma_{Fc}=\tau_{c}\sqrt{\frac{E_{p}}{G_{m}}}$ ( 27 )

which provides a relationship between film strength and molecular stiffness (*E_p_*), inter-molecular shear modulus (*G_m_*), and intermolecular shear strength (*τ_c_*). Comparing these equations to the Yoon theory for fibers composed of linear molecules, the film strength at infinite molecular weight is 12% lower compared to a fiber of infinite molecular weight. Since composite failure is governed by shear failure, and the linear molecular composite has a higher shear area density (more shear interfaces per unit volume), local shear stresses are slightly reduced compared to the film composed of platelet molecules.

If we consider finite molecular weights, then for the same molecular aspect ratio a 2D film will also have 12% lower failure strength compared to a linear polymer fiber (ratio of Eqn. (24) to Eqn. (14)). However, a molecular weight in a 2D polymer is proportional to *s*^2^, whereas in a linear polymer molecular weight is proportional to *s*. Therefore molecular weights required to achieve high mechanical properties in a 2D polymer film will need to be considerably larger than the molecular weights currently used in linear polymer fibers.

S5.3 Fiber and film stiffness and failure strain from shear lag theory

A key step in the shear lag solution for molecular composites is the assumption that the average platelet tensile stress is equivalent to the average global tensile stress. Under this assumption, then Eqn. (10) for a molecular fiber composite can be rewritten as

$\sigma=E_{f}\varepsilon$ ( 28 )

where the average fiber modulus *E_f_* is given by

$E_{f}=E_{r}\left(1-\frac{\tanh\left(ns\right)}{ns}\right)$ . ( 29 )

Similarly, for the molecular composite film, Eqn. (21) can be rewritten as

$\sigma=E_{F}\varepsilon$ ( 30 )

where the average fiber modulus *E_f_* is given by

$E_{F}=E_{p}\left(1-\frac{\tanh\left(ns\right)}{ns}\right)$ . ( 31 )

Equations (29) and (31) give estimates for effective fiber and film modulus as a function of molecular aspect ratio. The effective bulk moduli are reduced relative to the rod or platelet moduli because the reinforcement is only partially loaded during the shear development zones at the ends of the reinforcement.

Strain at failure for fibers can be calculated by re-arranging Eqn. (11):

$\varepsilon_{fc}=\frac{2\tau_{c}}{nE_{r}}\coth\left(ns\right)$ . ( 32 )

Similarly, for films the strain at failure can be calculated by re-arranging Eqn. (22):

$\varepsilon_{Fc}=\frac{\tau_{c}}{nE_{p}}\coth\left(ns\right)$ . ( 33 )

For infinite molecular weight,

$\varepsilon_{fc}=\frac{2\tau_{c}}{nE_{r}}$ ( 34 )

and

$\varepsilon_{Fc}=\frac{\tau_{c}}{nE_{p}}$ . ( 35 )

S5.4 Effect of orientation on film strength

Allen *et al.* ^24^ demonstrated that, for high performance fibers, failure was driven by shear failure, and slight misorientations could drive significant reductions in fiber strength. Using an analogue to fiber composite theory, they proposed that a fiber with a characteristic molecular orientation *θ* would have a strength *σ_fc_*(*θ*) related to the perfectly oriented fiber strength *σ_fc_* and the shear strength of the matrix *τ_c_* as

$\sigma_{fc}(\theta)=\left(\frac{1}{\sigma_{fc}^{2}}\cos^{4}\theta+\frac{1}{\tau_{c}^{2}}\sin^{2}\theta\cos^{2}\theta\right)^{-1/2}$ . ( 36 )

Using *σ_fc_* from Eqn. (14) in Eqn. (36) allows for a prediction of fiber strength as a function of both molecular weight and molecular orientation. Note that real fibers will have distributions of molecular weights and orientation angles, and appropriate weight averaging of the probability functions is necessary for such materials. Eqn. (36) can be directly applied for the case of a uniform molecular aspect ratio *s* and orientation angle *θ*.

For a film composed of 2D molecules, the analogous mis-oriented film strength would be

$\sigma_{Fc}(\theta)=\left(\frac{1}{\sigma_{Fc}^{2}}\cos^{4}\theta+\frac{1}{\tau_{c}^{2}}\sin^{2}\theta\cos^{2}\theta\right)^{-1/2}$ . ( 37 )

where *σ_Fc_* for finite molecular weight film is given by Eqn. (24).

S5.5 Effect of orientation on film stiffness

Northolt et al. ^25^ found that fiber modulus *E_f_* could be related to the characteristic molecular orientation according to

$E_{f}(\theta)\cong\left(\frac{1}{E_{f}}+\frac{\sin^{2}\theta}{2G_{m}}\right)^{-1}$ ( 38 )

where *E_f_* is the modulus of a fiber with uniform molecular alignment along the fiber axis, and *θ* is the characteristic molecular alignment relative to the fiber axis. The combined effects of orientation and molecular weight can be estimated by using Eqn. (29) for *E_f_*. This combined equation is

$E_{f}(\theta)\cong\left(\frac{1}{E_{r}}\frac{ns}{\left(ns-\tanh\left(ns\right)\right)}+\frac{\sin^{2}\theta}{2G_{m}}\right)^{-1}$ . ( 39 )

Assuming that the same orientation dependence relationships apply for 2D molecular films, we can estimate that

$E_{F}(\theta)\cong\left(\frac{1}{E_{F}}+\frac{\sin^{2}\theta}{2G_{m}}\right)^{-1}$ ( 40 )

where *E_F_* is the modulus of a film with uniform molecular alignment along the fiber axis, which for finite molecular weight ensembles is given by Eqn. (31). The combined equation is

$E_{F}(\theta)\cong\left(\frac{1}{E_{p}}\frac{ns}{\left(ns-\tanh\left(ns\right)\right)}+\frac{\sin^{2}\theta}{2G_{m}}\right)^{-1}$ . ( 41 )

Because the analysis is linear elastic, the strain at failure for mis-oriented fibers can be calculated as:

$\varepsilon_{fc}=\frac{\sigma_{fc}(\theta)}{E_{fc}(\theta)}$ . ( 42 )

Similarly, for films the strain at failure can be calculated as:

$\varepsilon_{Fc}=\frac{\sigma_{Fc}(\theta)}{E_{Fc}(\theta)}$ . ( 43 )

***S6. Sensitivity of shear lag analysis to values of δ and θ***

Figure S2 shows the limiting stiffness and strength for graphene, graphylene, graphamid, and PPTA as a function of orientation angle. The data was generated using Eqns. (36), (37), (38), and (40) (shear lag limits for infinite molecular weight), and molecular properties (extensional modulus, shear modulus, and shear strength) are identical to those used for the generating Fig. 4c-f. The results show that mechanical performance is highly orientation dependent, but that the trends are relatively consistent with orientation angle. One exception is the comparison of elastic modulus for graphene and graphamid; at very low angle (less than 8°), graphene exhibits higher stiffness, whereas at orientation angles above 8° graphamid exhibits a higher stiffness.

Figure S4 shows stiffness and strength as a function of molecular weight for graphene, graphylene, graphamid, and PPTA, for cases of *δ* = 5, 10, and 20. As *δ* increases (larger numbers of molecules per fundamental shear lag element), higher molecular weights are required to build strength and stiffness in the molecular ensemble. The property shifts as *δ* changes from 5 to 20 for all cases are less than an order of magnitude, supporting the use of this theory to predict order-of-magnitude behaviors in polymeric ensembles.

1. : Effect of orientation angle on a) elastic modulus and b) strength for graphene, graphylene, graphamid, and PPTA. For all cases, infinite molecular weight is assumed.

1. : Effect of molecular weight on a) stiffness, for *δ* =5 and 10, b) stiffness, for *δ* =10 and 20, (a) strength, for *δ* =5 and 10, a) and strength, for *δ* =10 and 20. For all cases, molecular orientation is assumed to be uniform at 10°.

***S7. Comparison of model predictions to experimental data for PPTA and graphene paper***

***PPTA***

Rao et al. ^26^ provide strength and stiffness values for five different grades of as-spun PPTA, and for PPTA yarns that have been subject to various thermomechanical treatments. Molecular orientation for each yarn is measured via x-ray diffraction. They find that applying heat with no tension leads to relaxation and increase in the molecular orientation angle, while applying heat with tension can reduce the molecular orientation angle for some grades of PPTA. Figure S5 shows that the modulus data shows a strong correlation with orientation angle, while strength shows a weaker dependence. Also shown in Fig. S5 are shear lag model predictions, using the equations and parameters as described in Section 2.3. Calculation results are shown for orientation angles from θ = 4-22°, and for molecular aspect ratios of *s* = 10-∞. The results show that the model underpredicts experimental modulus values at higher orientation angles, while predicting strength values within the scatter of the data.

Allen et. al^24^ hypothesized that fiber strength and modulus would both strongly correlate with molecular orientation, and therefore strength and modulus should correlate well to each other. They found that strength did not correlate well with initial fiber modulus (as measured at low strains), but that a strong correlation could be found with the "asympototic" fiber modulus (measured immediately before failure). Figure S6a shows a strength versus modulus correlation for shear lag model predictions, using the equations and parameters as described in Section 2.3. To create this plot, strength and modulus were calculated at orientation angles from θ = 0-30°, and for molecular aspect ratios of *s* = 10-∞, and then all results were assembled into a single data set of strength versus modulus. Figures S6b and S6c compare the model correlation with experimental data from Allen et al.^24^. The model predictions are within the data scatter for initial modulus data (Fig. S6b), while showing good correlation with the experimental data for asymptotic modulus (Fig. S6c).

Little data is available is the literature that correlates PPTA molecular weight with fiber mechanical properties. Further complicating such data sets is that all polymer fibers will possess a molecular weight distribution, for which distribution function weighting factors are best suited for representing ensemble behavior. Northolt et al.^22^ provide a limited data set of mechanical properties for PPTA at number-averaged molecular lengths of 60-83 nm (Table S8). Figure S7 plots this data, and compares it with shear lag model predictions for PPTA as a function of molecular length and at orientation angles of θ = 0-30°, in all cases assuming *δ* =10. Experimental modulus data tracks with model predictions for θ = 5°, suggesting that these particular PPTA fiber were highly oriented. Strength values are generally higher than model predictions, with best fit for orientation angles approaching θ = 0°, perhaps suggesting that additional orientation was induced by tensile loading. Reducing *δ* would also lead to better overlap between model and experimental data sets.

1. : Effect of orientation angle on model predictions for a) ensemble elastic modulus and b) ensemble strength for PPTA for different shear lag aspect ratios *s* = 10 - ∞. For all model values, *δ* =10. Experimental data for PPTA is from Rao et al.^26^.

1. : a) Correlation between ensemble strength and ensemble elastic modulus for PPTA, calculated over the range of θ = 0 - 30° for shear lag aspect ratios *s* = 10 - ∞. For all model values, *δ* =10. Comparison with experimental PPTA data from Allen et al.^24^, b) using initial experimental elastic modulus values and c) using asymptotic experimental modulus values.
2. : Experimental data for PPTA fiber elastic modulus and strength as a function of molecular length, from Northolt et al.^22^. Monomer weight is 238 g/mol, and monomer length is 1.3 nm. *z_n_* is the number of monomer units in the average molecule.

1. : Effect of molecular length on model predictions for ensemble a) elastic modulus and b) strength of PPTA, for θ = 0-10°. For all model values, *δ* =10. Experimental data for PPTA is from Northolt et al.^22^, as given in Table S8.

***Graphene paper***

One possible benchmark for the graphene ensemble predictions is "graphene paper" (GP) ^27,28^. GPs are formed by depositing graphitic flakes from solution, resulting in a paper with global stiffness and strength derived from interactions between the flakes. Two notable examples ^27,28^ both start with 50-100 µm sized (mean diameter) graphite flake, which is converted to graphite oxide in concentrated sulfuric acid, transfered to water and exfoliated via ultrasonication, converted back to graphene via reduction in hydrazine, and then filter-deposited, dried into a paper, and optionally heat treated to densify.

In principle, these GPs are ensembles of graphitic platelets, and therefore should obey the shear lag analysis developed in Section 2.3. However, the mechanical properties of the best performing GPs are relatively poor, achieving a stiffness of 40 GPa and a strength of 0.3 GPa compared to theoretical aligned limits of 1000 GPa and 6 GPa. The most likely non-ideality of these GPs is the interaction between platelets. The values for shear modulus and shear strength calculated via DFT in Section 2.2 are for closely-spaced graphene planes, approaching crystalline graphite spacings. These interactions will reduce drastically as the surfaces are spaced farther apart, or are less flat and parallel. It is likely that the assembly process for GPs leads to non-flat, non-close interactions due to trapped water and other impurities, as well as folds and wrinkles in the individual platelets. To simulate this effect, Fig. S8 shows elastic modulus and strength predictions for graphene ensembles, assuming δ = 10 and θ = 10°. For each case, the effective shear modulus and shear strength (isotropic graphene values from Table S4) are scaled by a factor λ, to approximate the effect of decreasing interactions in a poorly stacked ensemble. Also shown in the figure is the best-performing experimental data from Chen et al.^27^, using 78 µm as the mean platelet size (from manufacturer data). The results suggest that the inter-domain shear interactions are approximately 2-5× lower in graphene paper, compared to ideal inter-molecular interactions.

Another factor in GP performance could be the effective aspect ratio of individual shear lag domains. The current shear lag model has been implemented for the case of δ = 10, which would suggest that each platelet is 78 µm in diameter and 3.6 nm thick. It is possible that the GPs are formed from partially-exfoliated graphene flakes, or that the flakes agglomerate into thicker multi-layer domains during filtration. Figure 9 shows the effective mechanical properties of ensembles composed of 78 µm platelets with thicknesses ranging from 0.04-40 µm. Orientation angle is assumed to be uniformly θ = 10°, and there is no scaling of the intermolecular shear stress and modulus (λ=1). The results show that increasing platelet thickness, and reducing platelet aspect ratio, leads to poorer mechanical properties. Platelet thicknesses of 4-40 µm would result in mechanical behavior similar to observed mechanical properties in GP.

The true mechanics of the graphene paper system is likely strongly influenced by both weak inter-domain interactions, and imperfect exfoliation. Additional factors could include average orientation angles θ greater than 10°, and reduced molecular stiffness and strength due to damage from the oxidation, reduction, and exfoliation processes. Furthermore, the shear lag theory results suggest that careful stacking of clean and flat graphene sheets could produce graphene papers with significantly higher mechanical performance compared to existing experimental results.

1. : Effect of molecular length and interaction scaling factor λ on model predictions for a) elastic modulus and b) strength of graphene ensembles, for θ = 10° and δ = 10 . Experimental data for graphene paper is from Chen et al.^27^.

1. : Effect of molecular length and δ on model predictions for a) elastic modulus and b) strength of graphene ensembles, for θ = 10°. Experimental data for graphene paper is from Chen et al.^27^. Also shown for each curve is the effective shear lag domain thickness, *h*.

***S8. Details on fracture analysis***

Fracture simulations were performed using classical MD in the LAMMPS software package^29^. with the unmodified ReaxFF potential^30^. We benchmarked the ReaxFF potential with DFT simulations of graphamid in uniaxial tension, with excellent agreement between the predicted stiffness and strength of graphamid, as seen in Fig. S9. The parameters used for both simulations are outlined in the methodology section.

The strain, 2D stress and area-normalized fracture energy for the fracture simulations of graphene, graphylene and graphamid can be seen in Table S9. Graphene and graphylene fail at similar strains while graphamid has a significant increase in strain to failure between the 1L and 3L fracture simulations. The fracture energy of graphamid is higher than graphylene or graphene in the 1L, and 3-4× higher than graphylene or graphene in the 3L test. The curve fits of extrinsic stress concentration from Eqn. 6 (main body) can be seen in Fig. S10 and the results of the fits can be found in Table S8.

1. : a) Engineering stress/strain response for uniaxial tension of one layer graphamid in the 1NN direction as computed by molecular dynamics (MD) and density functional theory (DFT), b) unstrained graphamid domain and c) strained graphamid domain.
2. : Strain and stress at fracture for graphene, graphylene, and graphamid in 1L and 3: pre-crack configurations. Fracture energy is reported per unit area, *U_fracture_ / A*, where *A* = 10nm x 20 nm.

1. : Local y-component stress as a function of distance from the crack tip, r, and corresponding least-squares fit of equation (6) (dashed line) for a) graphene 1L, b) graphene 3L, c) GrE-2 1L, d) GrE-2 rL, e) graphamid 1L and f) graphamid 3L. The results of the curve fits are reported in Table S10.
2. : Extrinsic stress concentration factors and far field stress for graphene, graphylene, and graphamid in 1L and 3L pre-crack configurations.

***S9. Reduction in GO intermolecular shear strength relative to theoretical maximum***

The theoretical maximum shear strength for a hydrogen bond can be loosely estimated with the amount of energy it takes to break a bond, *E_O-H_* in the case of an O--H hydrogen bond, the distance at which the hydrogen bond breaks, *d_break_*, the equilibrium distance, *d_e_*, and the areal hydrogen bond density of the material, *ρ_H_*. Trigonometry yields the shear displacement relationship to be *d_shear_* = (*d_break_*^2^ – *d_e_*^2^)^1/2^ (Fig. S12) and the maximum force required to break a bond is *F_max_* =*E_O-H_* /*d_shear_*. Finally, maximum shear stress is related as *τ_max,c_* = *F_max_*·*ρ_H_*. With *E_O-H_* = 0.32eV/bond (5.13·10^-20^J/bond), *d_e_* = 0.2nm, *d_break_* = 0.4nm, *ρ_H_* = 7 bonds/nm^2^*, τ_max,c_* = 1.04GPa.

**Figure S12: Diagram of a single hydrogen bond failing under shear deformation for the estimation of theoretical maximum shear strength**

Consider two opposing GO surfaces, each randomly populated with 1/3 of available sites as epoxide, 1/3 as hydroxide, and 1/3 as unfunctionalized sites. In this scenario, the total functionalization is 66%, approaching the maximum experimentally achieved value of 70% functionalization, and the stoichiometric ratio of epoxides to hydroxides is ideally balanced. By simple probabilities, the likelihood of an epoxide finding a hydroxide on the opposing surface is 2/9. Therefore, only 22% of the available sites will form hydrogen bonds. Combining this percentage with other factors - the propensity to form intra-layer versus inter-layer bonds, and the significant corrugation of epoxide-functionalized GO - then the total population of hydrogen bonds is likely to be lower than 10% of the theoretically available maximum.

**Supplemental references**

1 Cadelano, E., Palla, P. L., Giordano, S. & Colombo, L. Nonlinear elasticity of monolayer graphene. *Physical review letters* **102**, 235502 (2009).

2 Phoenix, S. L. & Porwal, P. K. A new membrane model for the ballistic impact response and V 50 performance of multi-ply fibrous systems. *International Journal of Solids and Structures* **40**, 6723-6765 (2003).

3 Northolt, M. & Van Aartsen, J. On the crystal and molecular structure of poly‐(p‐phenylene terephthalamide). *Journal of Polymer Science Part C: Polymer Letters* **11**, 333-337 (1973).

4 Northolt, M. & Van Aartsen, J. in *Journal of Polymer Science: Polymer Symposia.* 283-296 (Wiley Online Library).

5 Gaymans, R., Tijssen, J., Harkema, S. & Bantjes, A. Elastic modulus in the crystalline region of poly (p-phenylene terephthalamide). *Polymer* **17**, 517-518 (1976).

6 Barton Jr, R. Paracrystallinity-modulus relationships in kevlar aramid fibers. *Journal of Macromolecular Science—Physics* **24**, 119-130 (1985).

7 Northolt, M. Tensile deformation of poly (p-phenylene terephthalamide) fibres, an experimental and theoretical analysis. *Polymer* **21**, 1199-1204 (1980).

8 Allen, S. & Roche, E. Deformation behaviour of Kevlar® aramid fibres. *Polymer* **30**, 996-1003 (1989).

9 Allen, S., Roche, E., Bennett, B. & Molaison, R. Tensile deformation and failure of poly (p-phenylene terephthalamide) fibres. *Polymer* **33**, 1849-1854 (1992).

10 Deteresa, S., Allen, S., Farris, R. & Porter, R. Compressive and torsional behaviour of Kevlar 49 fibre. *Journal of Materials Science* **19**, 57-72 (1984).

11 Allen, S. Stress-coupling phenomena in anisotropic fibres. *Polymer* **29**, 1091-1094 (1988).

12 Fielding-Russell, G. S. Vol. 41 861-864 (SAGE Publications Ltd 1 Olivers Yard, 55 City Road, London EC1Y 1SP, England, 1971).

13 Tashiro, K., Kobayashi, M. & Tadokoro, H. Elastic moduli and molecular structures of several crystalline polymers, including aromatic polyamides. *Macromolecules* **10**, 413-420 (1977).

14 Ohta, T. Review on processing ultra high tenacity fibers from flexible polymer. *Polymer Engineering & Science* **23**, 697-703 (1983).

15 Termonia, Y. & Smith, P. Theoretical study of the ultimate mechanical properties of poly (p-phenylene-terephthalamide) fibres. *Polymer* **27**, 1845-1849 (1986).

16 Crist, B. The ultimate strength and stiffness of polymers. *Annual Review of Materials Science* **25**, 295-323 (1995).

17 Rutledge, G. & Suter, U. Calculation of Mechanical Properties of Poly (p-phenylene terephthalamide) by Atomistic Modelling. *Polymer* **32**, 2179-2189 (1991).

18 Grujicic, M. *et al.* Filament-level modeling of aramid-based high-performance structural materials. *Journal of materials engineering and performance* **20**, 1401-1413 (2011).

19 Cox, H. The elasticity and strength of paper and other fibrous materials. *British journal of applied physics* **3**, 72 (1952).

20 Piggott, M. *Load bearing fibre composites*. (Springer Science & Business Media, 2002).

21 Yoon, H. Strength of fibers from wholly aromatic polyesters. *Colloid and Polymer Science* **268**, 230-239 (1990).

22 Northolt, M., Den Decker, P., Picken, S., Baltussen, J. & Schlatmann, R. The tensile strength of polymer fibres. *Polymeric and Inorganic Fibers*, 1-108 (2005).

23 Tyson, W. & Davies, G. A photoelastic study of the shear stresses associated with the transfer of stress during fibre reinforcement. *British Journal of Applied Physics* **16**, 199 (1965).

24 Allen, S. R., Roche, E. J., Bennett, B. & Molaison, R. Tensile deformation and failure of poly(p-phenylene terephthalamide) fibres. *Polymer* **33**, 1849-1854, doi:http://dx.doi.org/10.1016/0032-3861(92)90483-D (1992).

25 Northolt, M. & Hout, R. Elastic extension of an oriented crystalline fibre. *Polymer* **26**, 310-316 (1985).

26 Rao, Y., Waddon, A. & Farris, R. The evolution of structure and properties in poly (p-phenylene terephthalamide) fibers. *Polymer* **42**, 5925-5935 (2001).

27 Chen, H., Müller, M. B., Gilmore, K. J., Wallace, G. G. & Li, D. Mechanically strong, electrically conductive, and biocompatible graphene paper. *Advanced Materials* **20**, 3557-3561 (2008).

28 Ranjbartoreh, A. R., Wang, B., Shen, X. & Wang, G. Advanced mechanical properties of graphene paper. *Journal of Applied Physics* **109**, 014306 (2011).

29 Plimpton, S. Fast Parallel Algorithms for Short-Range Molecular Dynamics. *Journal of Computational Physics* **117**, 1-19, doi:http://dx.doi.org/10.1006/jcph.1995.1039 (1995).

30 Chenoweth, K., van Duin, A. C. T. & Goddard, W. A. ReaxFF Reactive Force Field for Molecular Dynamics Simulations of Hydrocarbon Oxidation. *The Journal of Physical Chemistry A* **112**, 1040-1053, doi:10.1021/jp709896w (2008).
